# Rehabilitation of Lost Fingers Using Silicone Prostheses: A Case Series

**DOI:** 10.7759/cureus.111570

**Published:** 2026-06-26

**Authors:** Ram B Basany, Aashrith C Reddy, Kovela S Goud, Mukesh Kistamolla, Anam Chandrasekar

**Affiliations:** 1 Prosthodontics, SVS Institute of Dental Sciences, Telangana, Mahbubnagar, IND; 2 Prosthodontics, Rajiv Gandhi University of Health Sciences, Shivamogga, IND

**Keywords:** case series, cosmetic rehabilitation, custom-made prosthesis, digit rehabilitation, extraoral maxillofacial prosthesis, finger prosthesis, partial hand defect, prosthodontic rehabilitation, prosthodontic rehabilitation and maxillofacial prosthesis, silicone prosthesis

## Abstract

Traumatic hand injuries can have a profound impact on an individual's physical function, psychological well-being, and quality of life. Loss of fingers or hand structures may result from trauma, congenital anomalies, infections, or other pathological conditions, leading to significant impairment in hand dexterity, daily activities, occupational performance, and social interactions.

Prosthetic rehabilitation serves as an effective solution for restoring the appearance and functionality of the affected hand. Custom-designed hand prostheses not only improve aesthetics but also help patients regain confidence, independence, and social acceptance. Although prosthetic devices may not completely restore normal hand function, they play a vital role in enhancing the patient's overall quality of life.

With appropriate clinical expertise, careful treatment planning, and a compassionate approach, healthcare professionals can successfully rehabilitate individuals with hand defects. This article presents our efforts in designing and fabricating a custom hand prosthesis aimed at restoring aesthetics, improving patient confidence, and facilitating reintegration into daily life.

## Introduction

The human hand is a remarkably complex organ, indispensable for a vast range of activities from fine motor control, such as writing and intricate manipulation, to gross motor tasks, such as grasping. Beyond its mechanical capabilities, the hand plays a vital role in non-verbal communication and self-expression, significantly influencing an individual's self-esteem and social confidence.

Traumatic injury remains the predominant etiology of digit amputations globally, with crush injuries, workplace machinery accidents, and falls being common injuries.

Thermal injuries, such as extensive burns, are particularly devastating because of their comprehensive tissue involvement. Epidemiological data consistently report a higher prevalence of traumatic finger and thumb amputations (71%-79%), predominantly affecting young adults compared to older individuals, correlating with occupational and lifestyle exposure risks [1].

Management options for partial hand loss vary according to the extent of injury, residual stump condition, and patient expectations. While microsurgical replantation and reconstructive surgeries are often considered for anatomical restoration, their feasibility and long-term success are limited by factors such as vascular integrity, contamination, and tissue viability. In recent years, advanced technological solutions such as myoelectric and bionic prostheses have broadened rehabilitation possibilities [2]. However, when surgical reconstruction or bionic integration is not feasible or economically accessible, prosthetic rehabilitation using custom silicone prostheses remains a highly effective alternative [3].

This article highlights the efficacy of contemporary prosthodontic techniques in restoring aesthetic form. Two illustrative clinical examples are presented: a 36-year-old man with isolated traumatic thumb loss rehabilitated with a custom silicone thumb prosthesis and a 46-year-old woman with extensive fire-related loss of multiple fingers managed with a glove-type silicone prosthesis. Both cases demonstrate the potential of individualized silicone restorations to recreate natural appearance, enhance self-confidence, and improve function and social reintegration.

## Case presentation

Case 1

**Figure 1:**
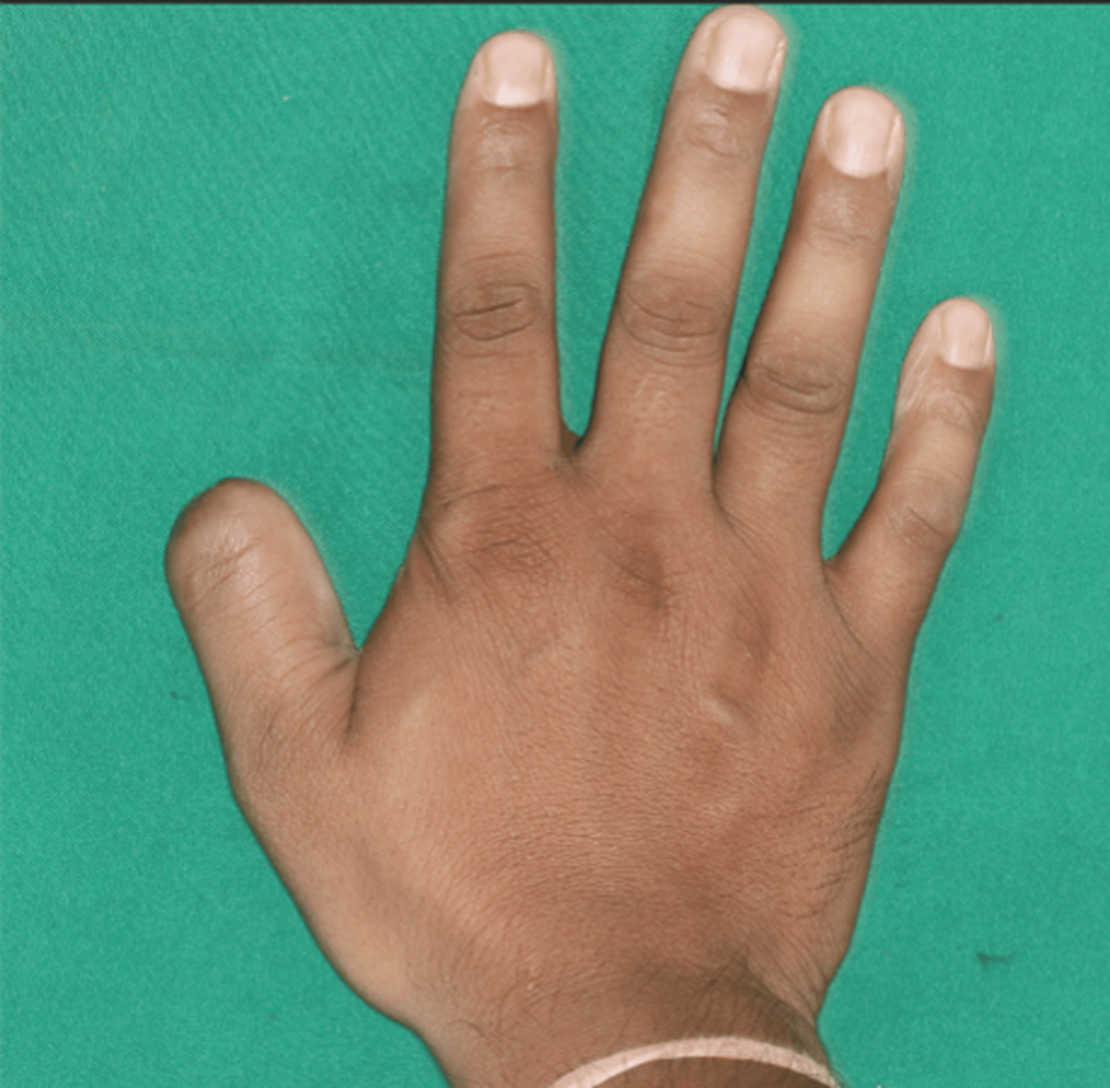
Dorsal view of the right hand showing the healed thumb stump

**Figure 2:**
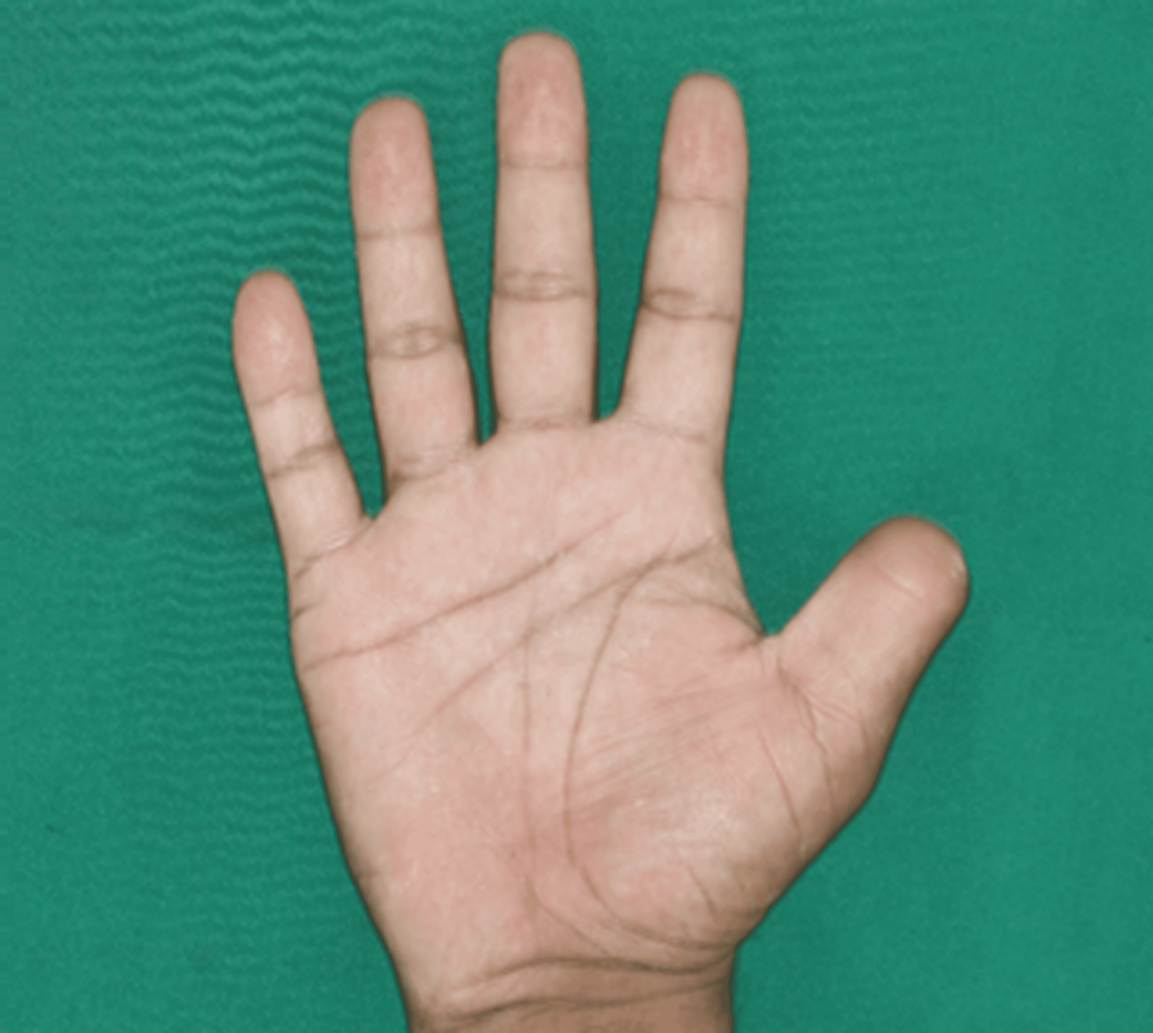
Ventral view of the right hand showing the healed thumb stump

A 36-year-old male patient reported to the Department of Prosthodontics, SVS Institute of Dental Sciences, Mahbubnagar, Telangana, with a loss of the right thumb. On clinical examination, the thumb was absent distal to the metacarpophalangeal joint. The trauma site demonstrated a rounded, well-contoured stump with adequate soft tissue coverage, located at the level of the metacarpal head. Clinical examination of the hand from the dorsal and ventral aspects revealed a well-healed residual stump with no signs of secondary infection or compromised tissue, as shown in Figure 1 and Figure 2.

Impression Technique

**Figure 3:**
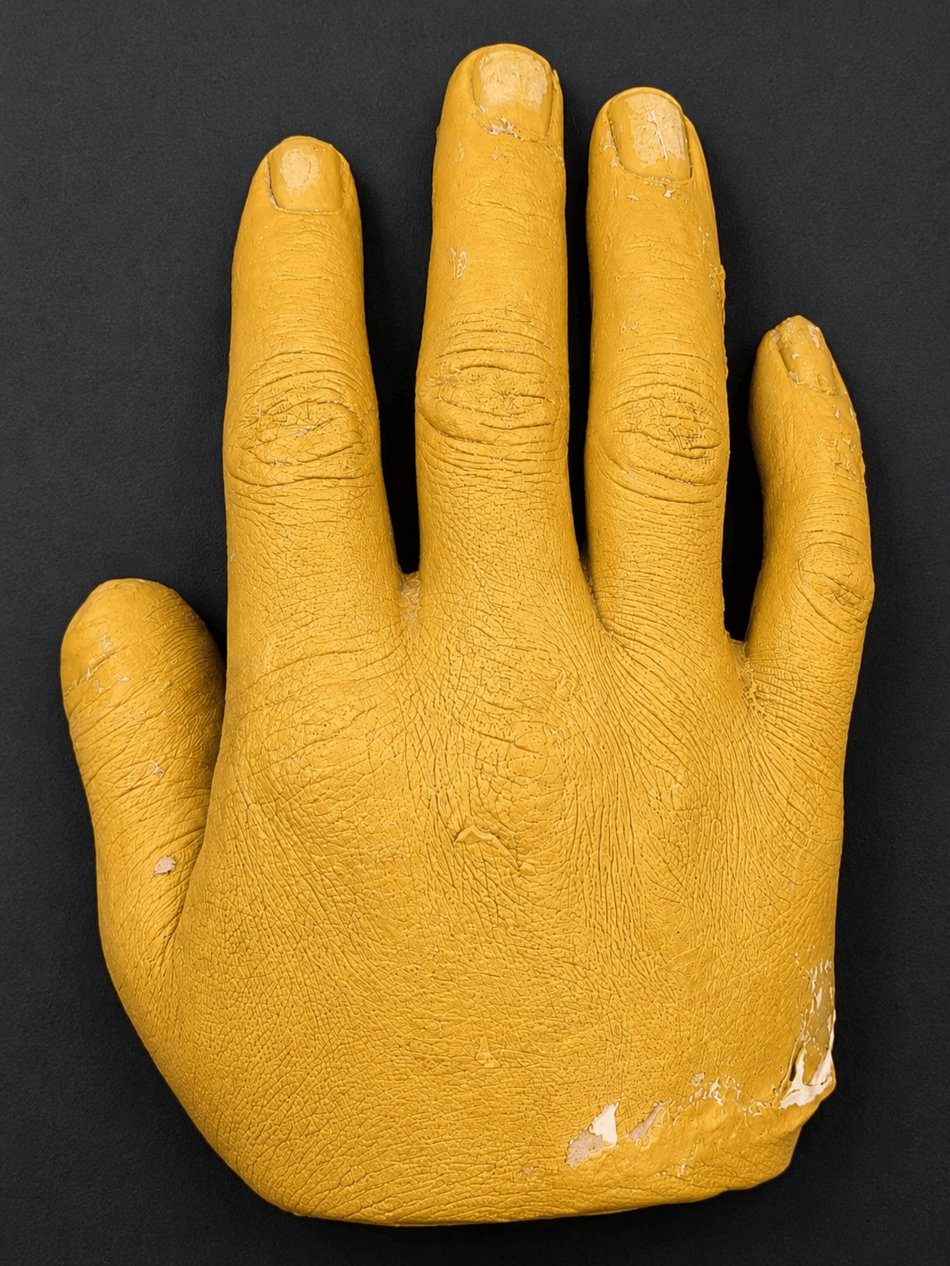
Master cast obtained from the hand impression

The patient's right hand was stabilized on a flat surface in a relaxed functional position, and a thin layer of petroleum jelly was applied to prevent adherence of the impression material. Irreversible hydrocolloid (Imprint; Dental Products of India Ltd., Mumbai, India) was mixed and carefully applied over the hand to record all surface details, followed by layering of wet gauze and dental plaster type II (Kaldent, Mumbai, India) over it to stabilize the material and prevent distortion. After setting, the impression was gently removed. A similar impression of the contralateral hand was made to serve as a guide for wax pattern sculpting. The impressions were poured in dental stone type III (Kalstone, Mumbai, India) to obtain a working master cast. Impression of the defect thumb and the contralateral thumb were made in addition silicone. Impression of the defect thumb was poured in type III stone, while the contralateral thumb impression was used to make a wax shell, as shown in Figure 3.

Wax Sculpting

**Figure 4:**
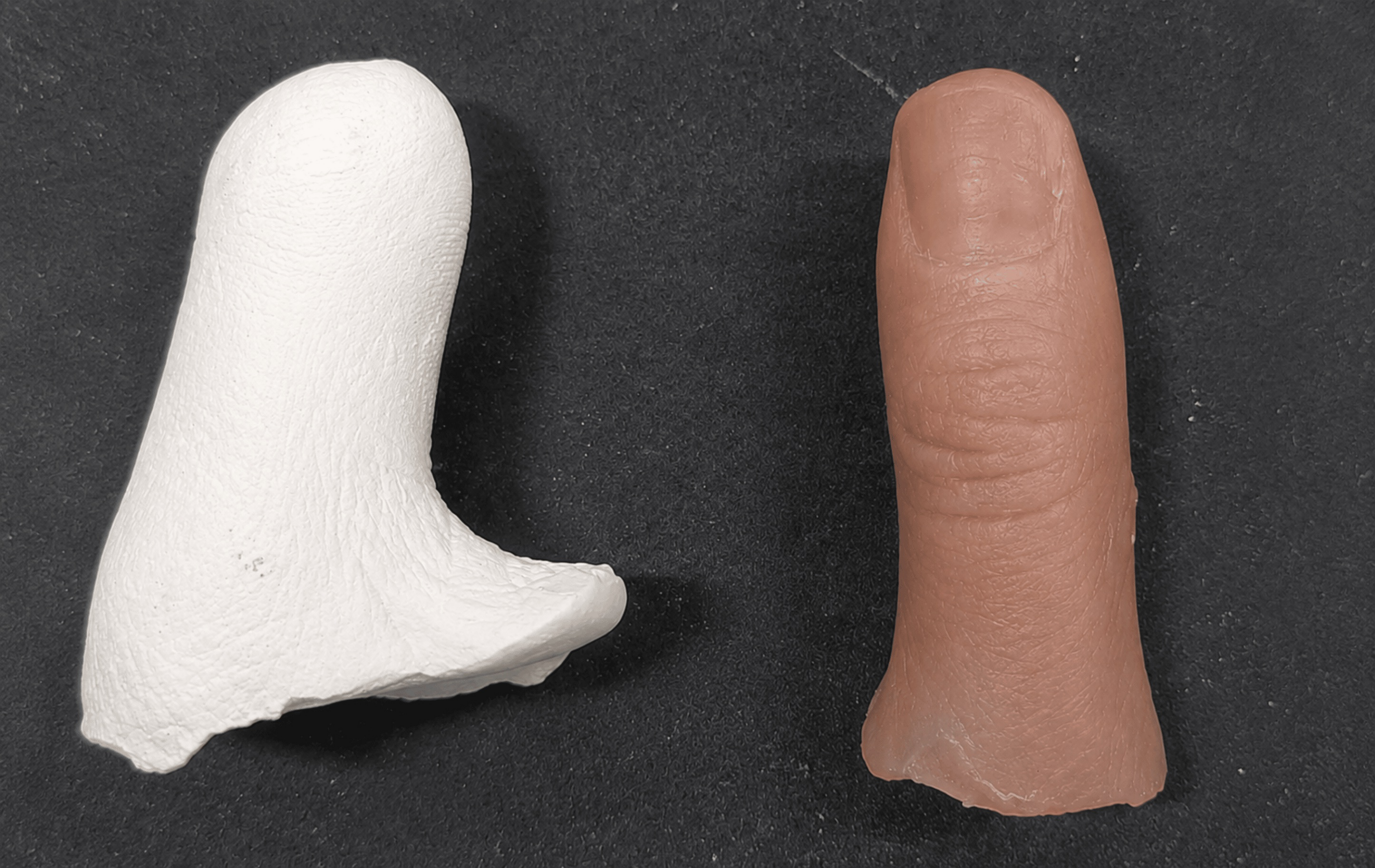
Wax pattern of the thumb fabricated on the master cast demonstrating accurate reproduction of anatomical contours, surface details, and morphology in comparison with the reference model

Wax sculpting of the thumb was performed on the master cast using the contralateral thumb as a reference for dimensions and surface anatomy. To obtain a hollow wax pattern, molten wax was poured into the impression and removed 4-5 times until the desired form and thickness were achieved. The final pattern reproduced the natural contours, nail bed, and morphology for lifelike aesthetics and symmetry, as shown in Figure 4.

Stump Modification on Working Model

To provide a snug fit for the prosthesis, approximately 0.5-1 mm of the stump surface on the working model was uniformly reduced. This ensured that the final silicone prosthesis would flex and stretch slightly during insertion and removal, thereby enhancing retention and comfort. Nail impressions were made using addition silicone, and customized artificial nails were fabricated with heat-cured acrylic resin to replicate natural color, translucency, and contour. These nails were integrated into the wax pattern to enhance realism.

Flasking and Dewaxing

**Figure 5:**
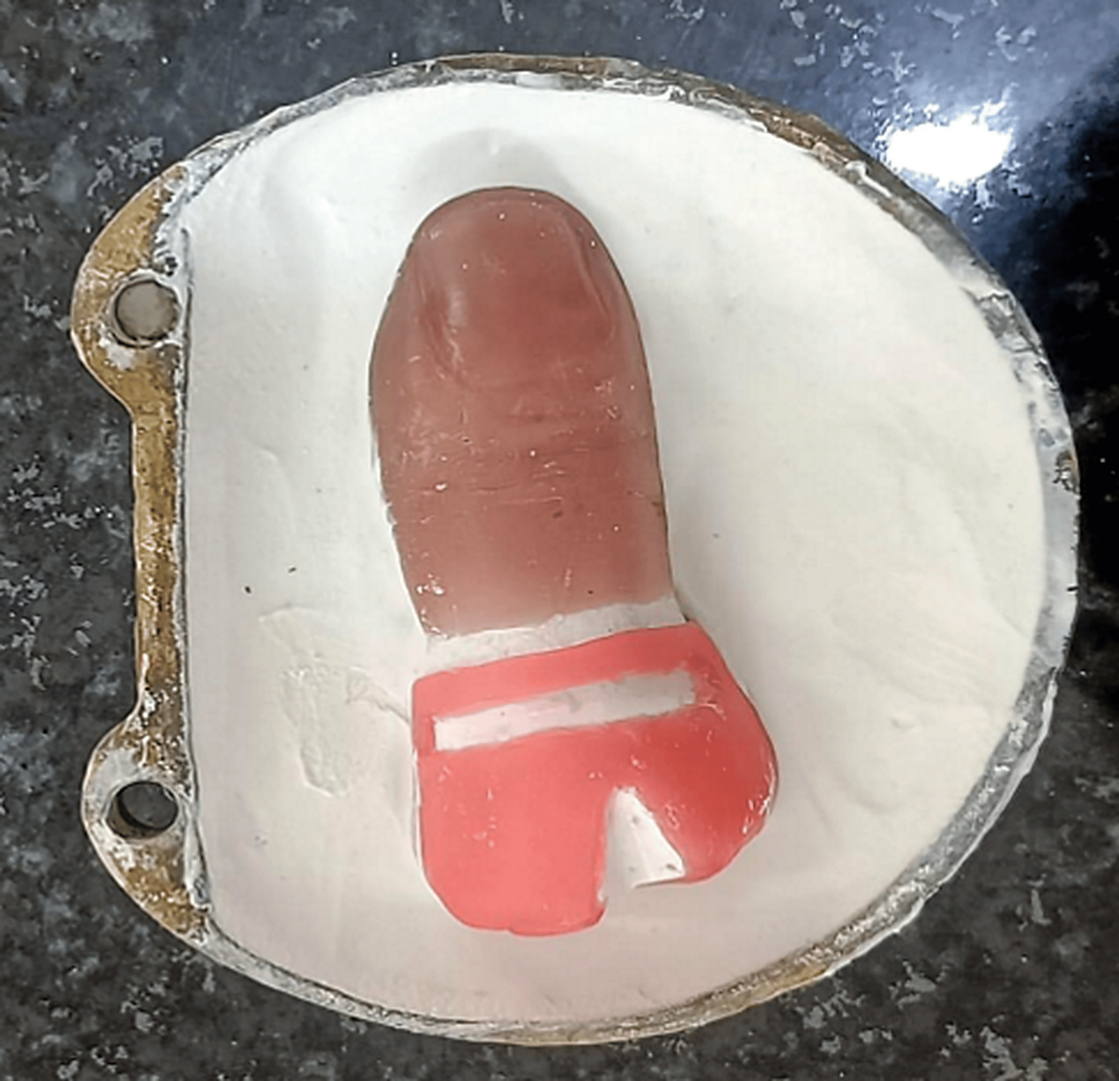
Invested wax pattern of the thumb in a three-part dental flask

Prior to investing, a separating medium was applied to the gypsum mold surfaces (F-901 Separating Film, Mumbai, India), followed by spraying a thin coat of releasing spray. Once the wax-up was approved, the pattern was invested in a number 9 dental flask using dental stone type III (Kalstone, Mumbai, India), employing a three-part mold technique to facilitate accurate retrieval. The flask was placed in boiling water to facilitate dewaxing, leaving behind a negative mold space corresponding to the sculpted fingers, as shown in Figure 5.

Processing of Silicone and Characterization

**Figure 6:**
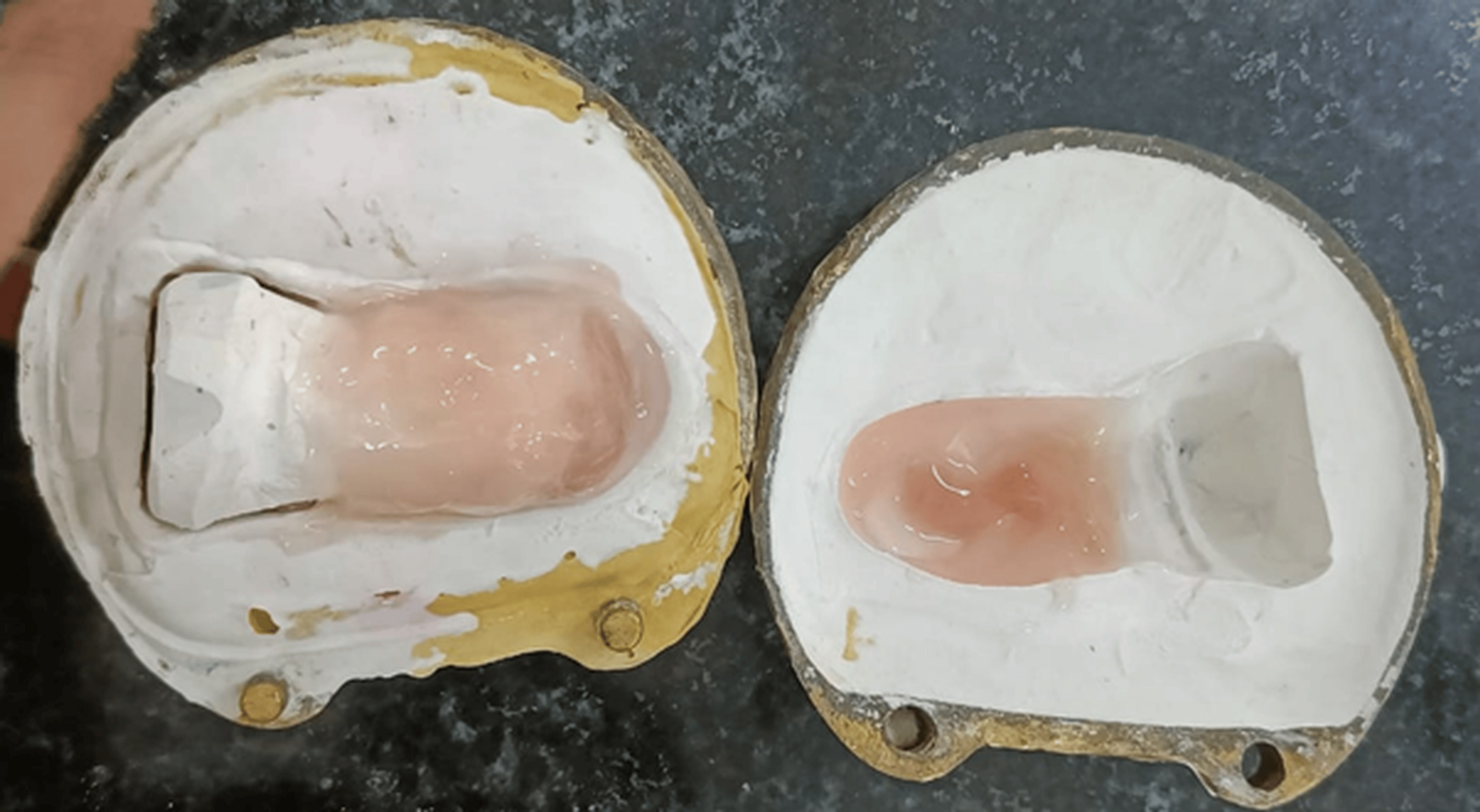
Packing of intrinsically stained medical-grade silicone elastomer into the three-part mold prior to curing

The mold was packed with medical-grade silicone elastomer (Part A of Part B of the M511, Technovent, Bridgend, UK). Intrinsic pigments (Technovent, Bridgend, UK) were incorporated into the silicone to achieve the desired base shade, closely matching the patient's natural tones. After thorough mixing, the intrinsically stained silicone was carefully packed into the prepared three-part mold, ensuring complete filling of all anatomical details and the absence of air entrapment, as shown in Figure 6.

The flask was then securely closed, and the silicone was cured under pressure as per the manufacturer's recommendations. Following curing, the mold was carefully separated, and the prosthesis was retrieved and trimmed, and excess silicone flash was removed using silicone trimming wheels mounted on a mandrel (5125 Silicone Trimming Wheels and Mandrel Set; Technovent, Bridgend, UK).

Extrinsic stains (Xtrinsic silicone inks; Spectromatch Ltd., Hampton, UK) were applied to replicate subtle variations in skin hue, surface texture, and pigmentation.

**Figure 7:**
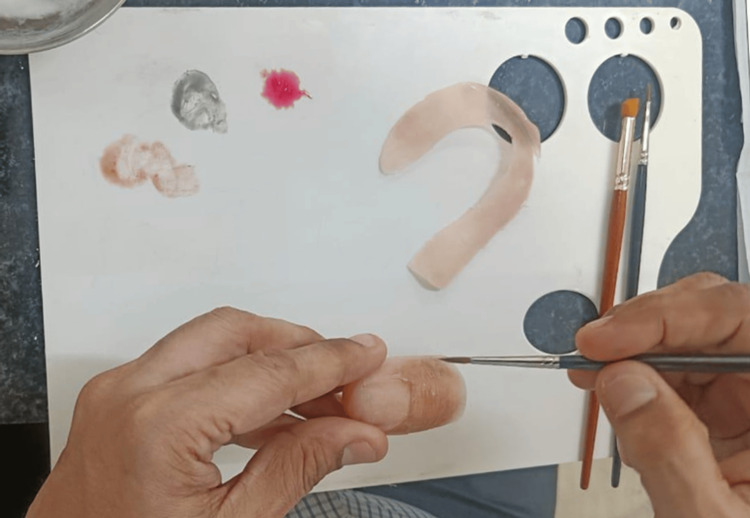
Extrinsic staining

The completed prosthesis was then tried on the patient's right hand, demonstrating good adaptation, comfort, and aesthetic harmony with the contralateral hand, as illustrated in Figure 7.

Final Insertion

**Figure 8:**
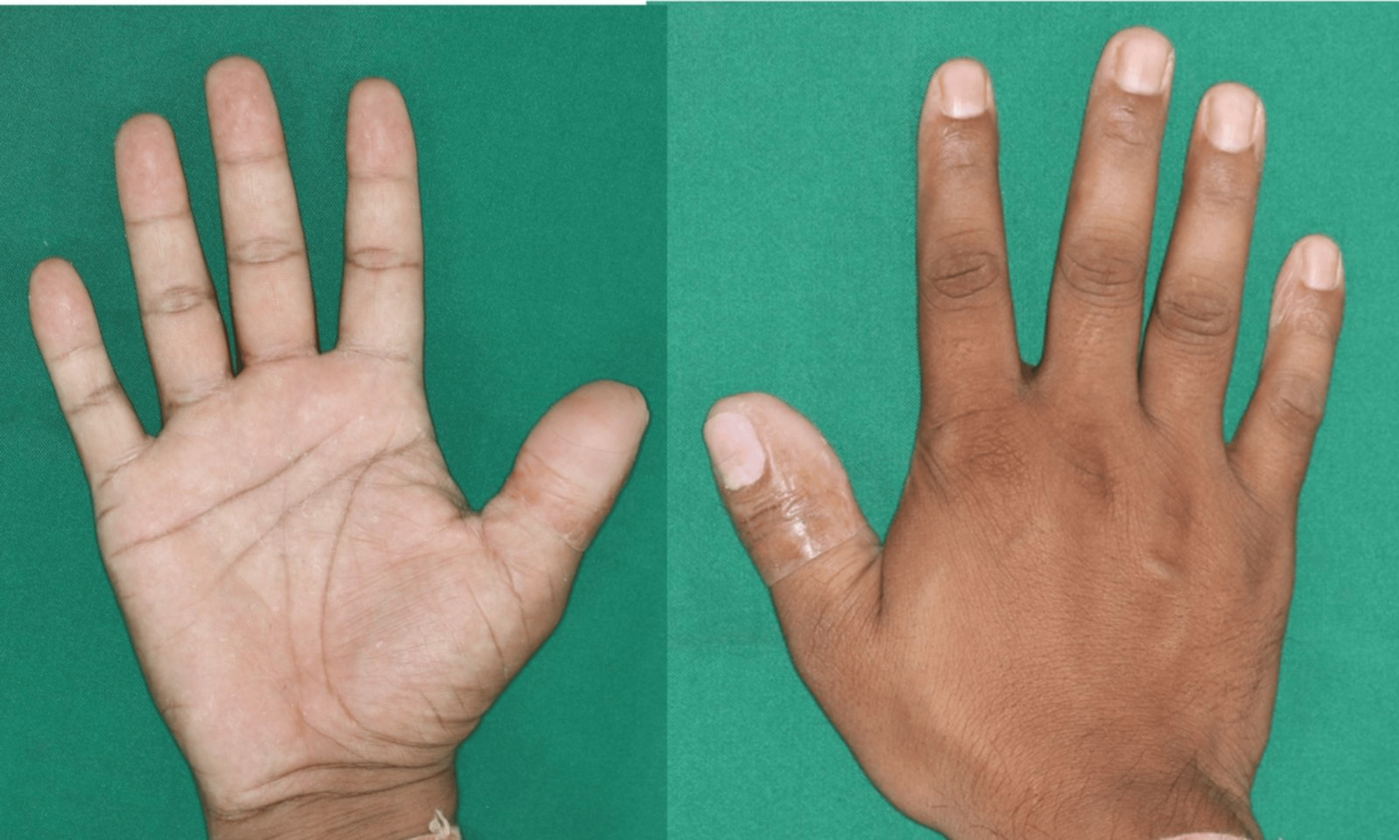
Final prosthesis after insertion on the patient's thumb

The final prosthesis was placed on the patient's hand. The patient was instructed about the use and maintenance of the prosthesis. The patient was advised to clean the prosthesis daily with mild soap and lukewarm water, pat it completely dry, and store it in a ventilated container away from heat and sunlight. Excessive bending, stretching, or contact with oils should be avoided. Periodic follow-up was recommended to check fit, color, and surface condition for timely adjustments. The final prosthetic outcome and aesthetic integration with the hand are shown in Figure 8.

Case 2

A 46-year-old female patient reported to the Department of Prosthodontics, SVS Institute of Dental Sciences, Mahbubnagar, Telangana, with a history of traumatic injury to the right hand.

**Figure 9:**
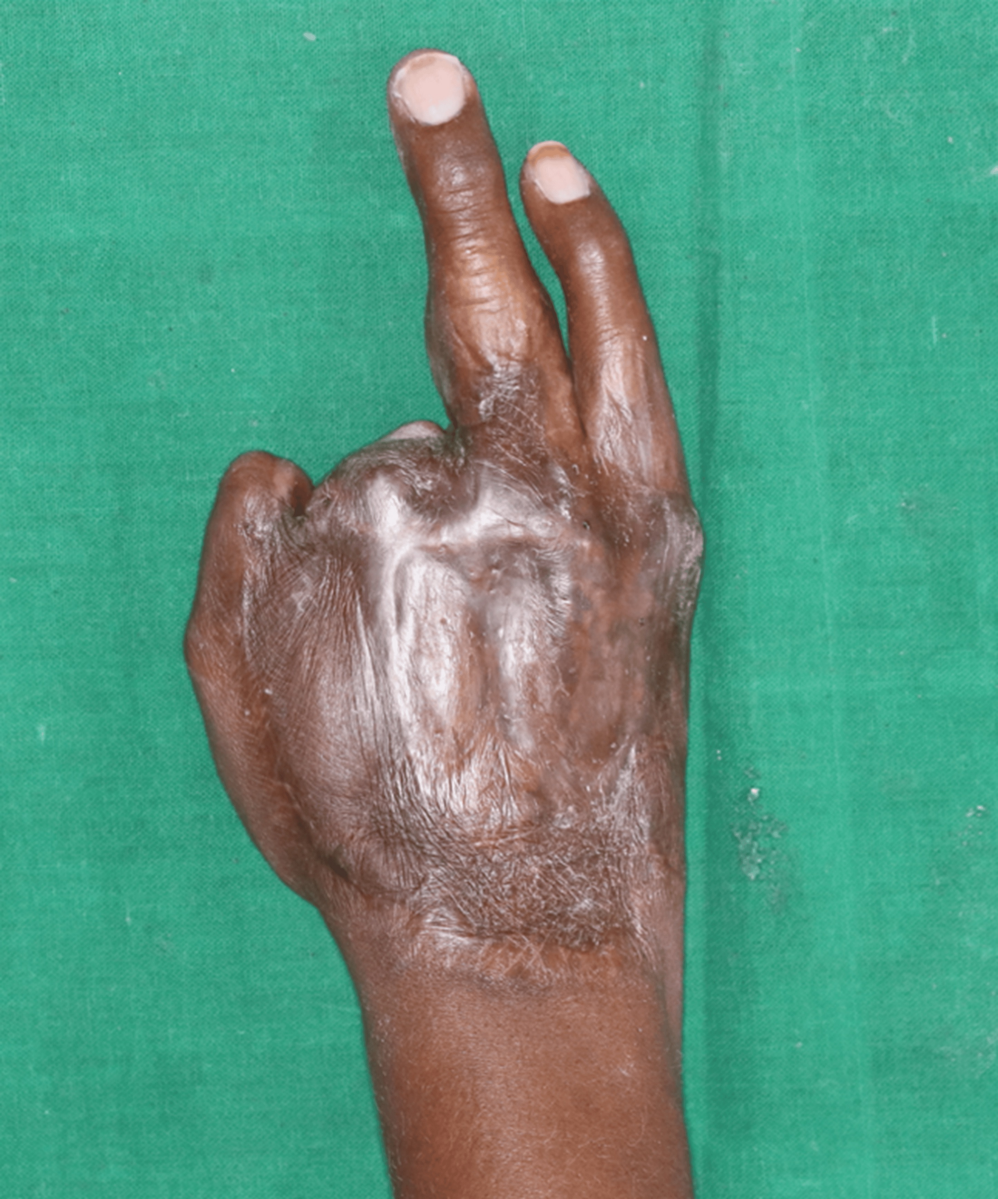
Dorsal view

Clinical examination of the dorsal aspect of the right hand revealed absence of the thumb, index, and middle fingers, with only the ring and little fingers retained. The dorsal surface demonstrated hypertrophic scarring and areas of skin graft take with a smooth but irregular surface. The soft tissues appeared stable, with no evidence of ulceration or active infection, as shown in Figure 9.

**Figure 10:**
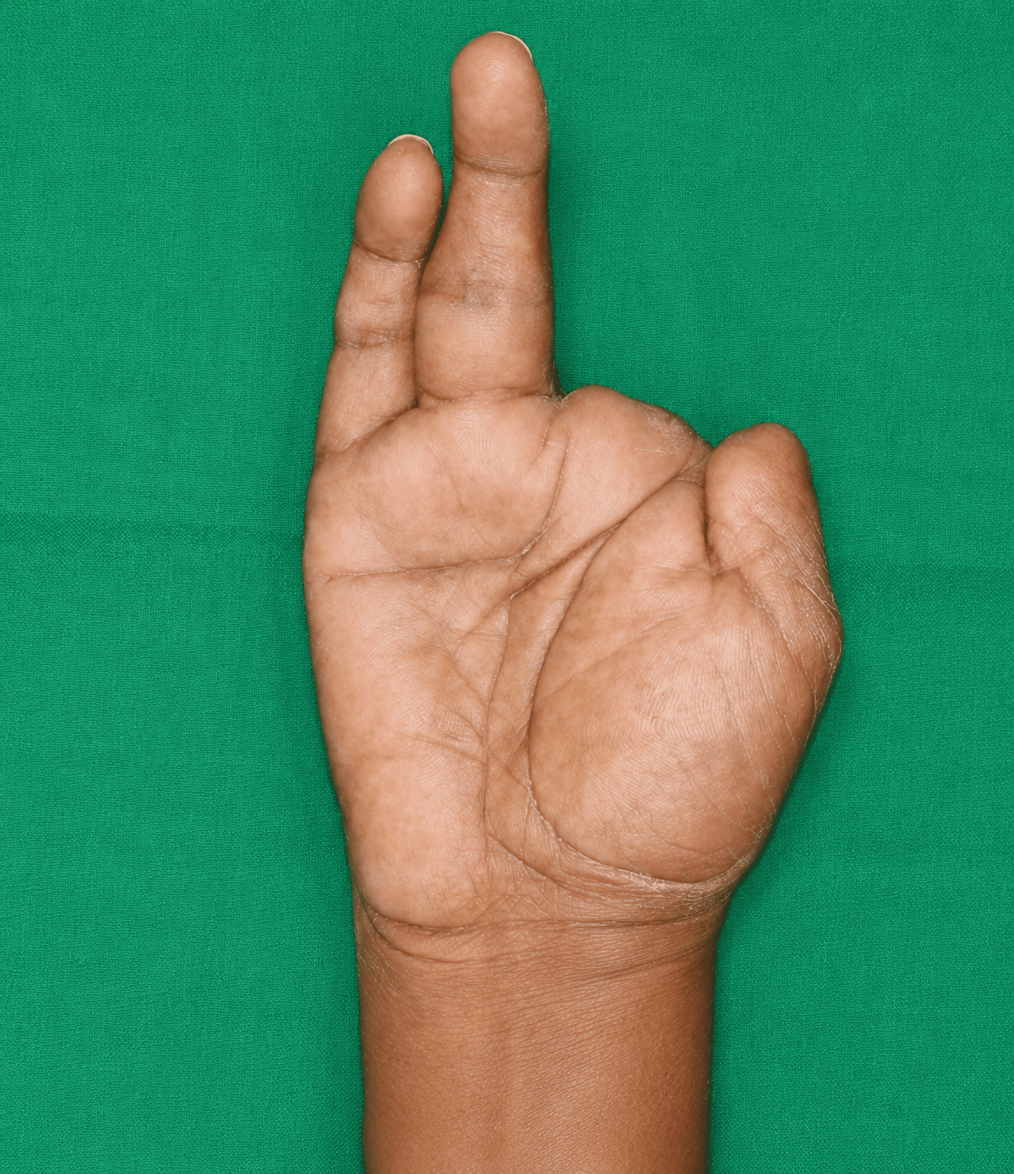
Ventral view

Clinical examination of the palmar aspect revealed amputation of the thumb, index, and middle fingers, while the ring and little fingers remained intact. The palmar skin demonstrated grafted areas with contracture bands. Although the patient had adapted to daily activities using the preserved ring and little fingers, fine manipulation and grip strength remained significantly compromised, as shown in Figure 10.

Impression Technique

**Figure 11:**
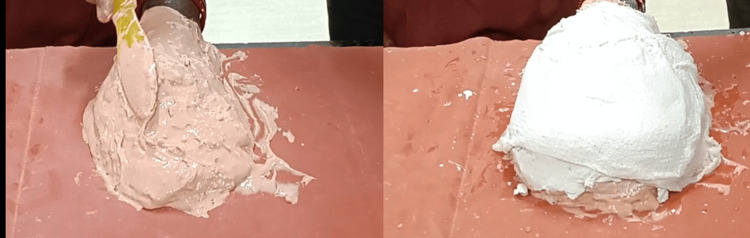
Hand impression-making procedure

The patient's right hand was stabilized on a flat table surface in a relaxed functional position. Petroleum jelly was applied over the skin to prevent adherence of the material. Irreversible hydrocolloid (Imprint; Dental Products of India Ltd., Mumbai, India) was mixed according to the manufacturer's instructions and carefully poured over the dorsal side; it was immediately reinforced by placing layers of wet gauze over the alginate surface, followed by layering of dental plaster type II (Kaldent, Mumbai, India) for backing to stabilize the material and prevent distortion. After the plaster had set, the hand was repositioned, and the ventral (palmar) aspect was similarly recorded, ensuring continuity with the previously formed dorsal impression, as shown in Figure 11.

Care was taken to avoid air entrapment. The impression was allowed to set while the hand remained immobile. After complete setting, the impression was carefully separated. Impression of the opposite hand was also made in a similar fashion, which served as a guide in the fabrication of the wax pattern. The impression was then poured with type III dental stone (Kalstone, Mumbai, India), and thus, a positive replica was obtained.

Wax Pattern Fabrication

**Figure 12:**
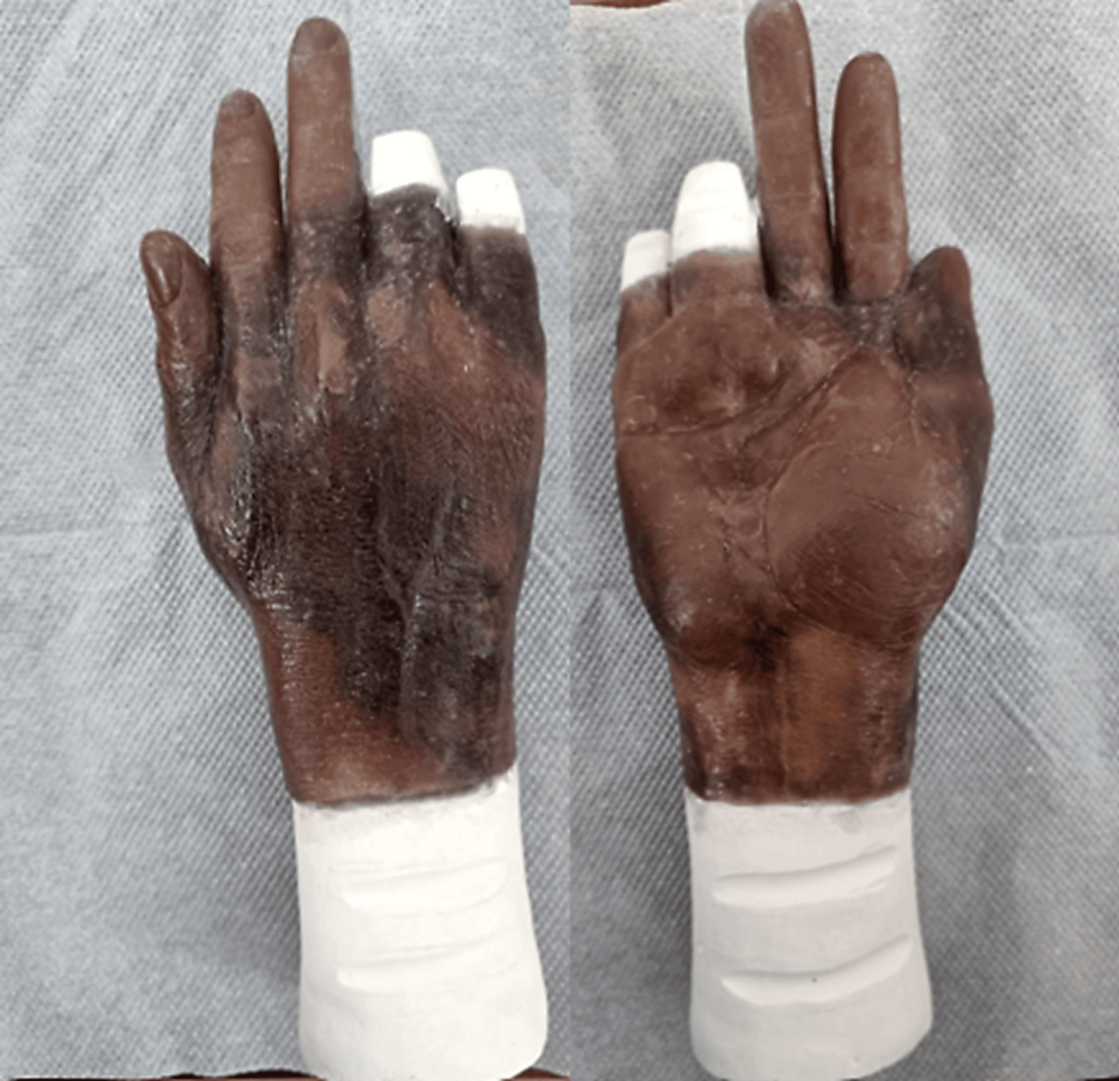
A wax pattern of the missing fingers was sculpted on the modified master cast using the contralateral hand as a guide to reproduce natural anatomy and appearance

The cast obtained from the impression was used for sculpting the preliminary prosthesis. The contralateral (left) hand was taken as a reference to reproduce the natural anatomy, contour, and proportions. Before initiating the wax pattern fabrication, approximately 2 mm of the cast surface in the defect region was uniformly scraped to ensure a snug fit of the final prosthesis. Modelling wax was adapted and sculpted over the cast to simulate the missing thumb, index, and middle fingers. Nail impressions were made using addition silicone material (Zhermack, Badia Polesine, Italy), and customized artificial nails were fabricated separately with heat-cured acrylic resin to replicate natural color, contour, and translucency. Special attention was given to maintain symmetry, finger length, and natural hand morphology to achieve an aesthetically acceptable result, as illustrated in Figure 12.

Flasking and Dewaxing

**Figure 13:**
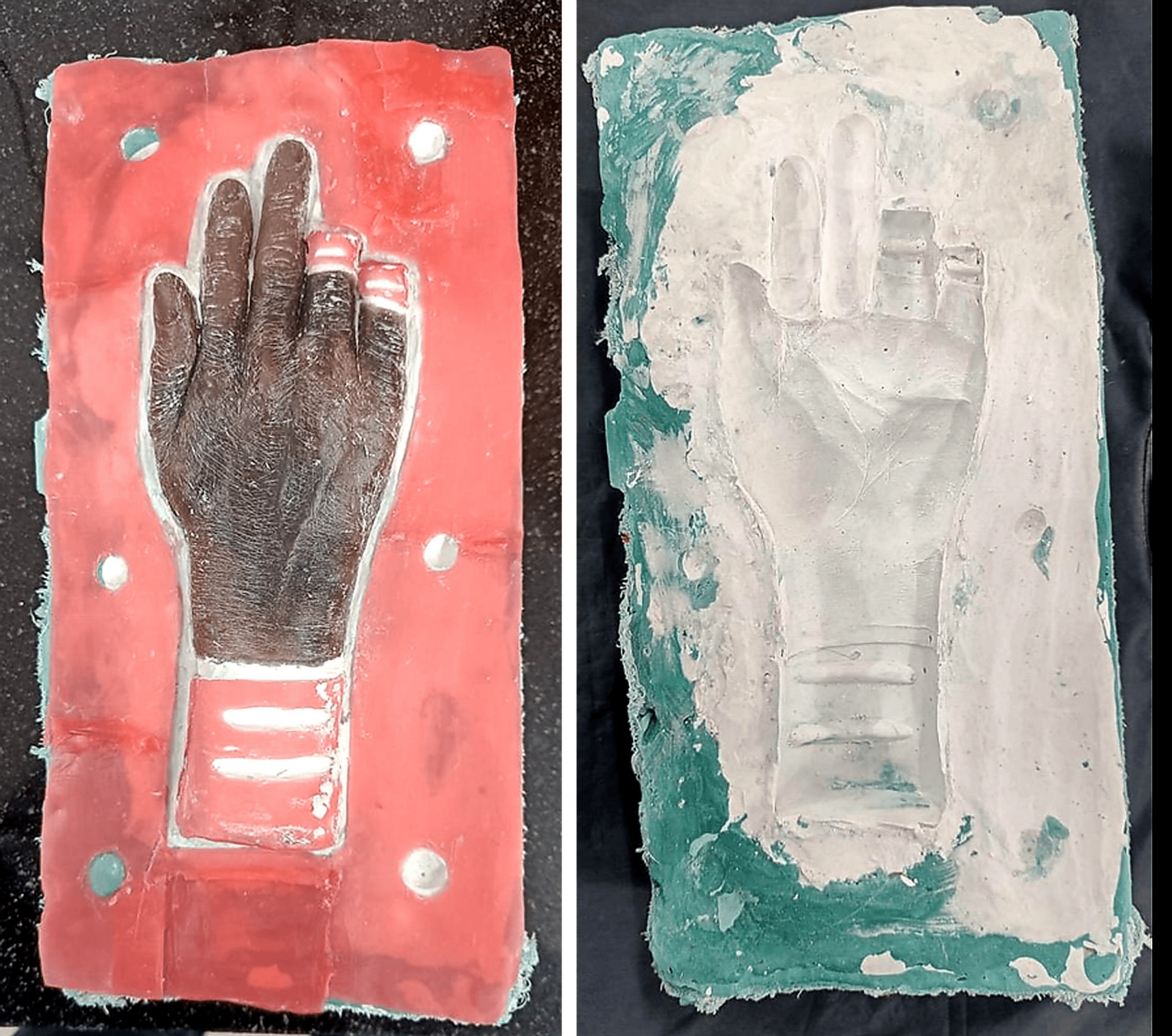
The approved wax pattern was flasked in dental stone using a three-part mold technique, followed by dewaxing to create the mold space for prosthesis fabrication

Once the wax-up was approved, the wax pattern was invested in a dental flask using dental stone. A three-part mold technique was employed for flasking to ensure accurate reproduction and facilitate easy retrieval of the prosthesis without distortion. After the stone had set, the flask was placed in boiling water to facilitate dewaxing, leaving behind a negative mold space corresponding to the sculpted fingers, as shown in Figure 13.

Processing of Silicone and Characterization

**Figure 14:**
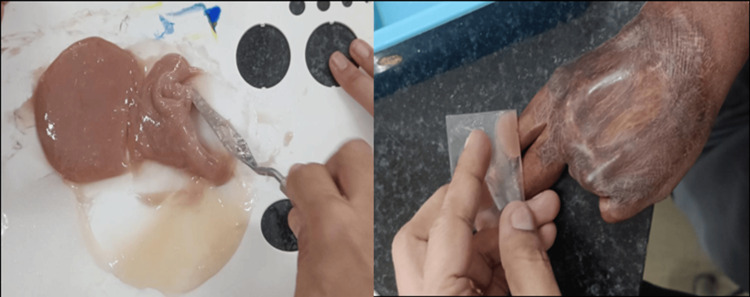
Medical-grade silicone was intrinsically stained to match the patient's skin tone and packed into the three-part mold, ensuring accurate reproduction of anatomical details

The mold was packed with medical-grade silicone elastomer (Part A of Part B of the M511, Technovent, Bridgend, UK). Intrinsic pigments (Technovent, Bridgend, UK ) were incorporated into the silicone to achieve the desired base shade, closely matching the patient's natural tones. After thorough mixing, the intrinsically stained silicone was carefully packed into the prepared three-part mold, ensuring complete filling of all anatomical details and the absence of air entrapment, as shown in Figure 14.

**Figure 15:**
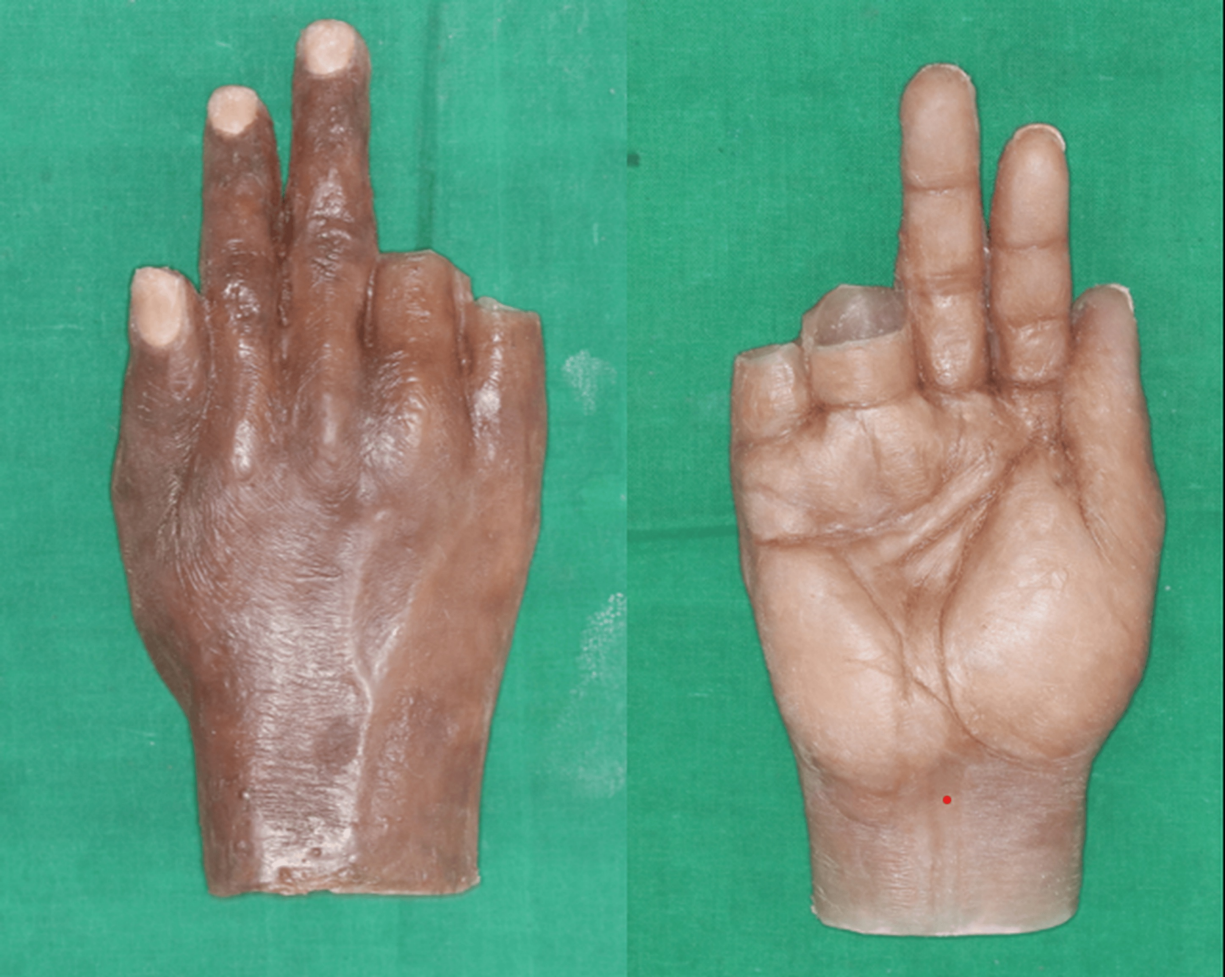
Extrinsic stains were applied to enhance the natural appearance of the silicone prosthesis

The flask was then securely closed, and the silicone was cured under pressure as per the manufacturer's recommendations. Following curing, the mold was carefully separated, and the prosthesis was retrieved, trimmed, and finished to refine the margins. Extrinsic stains (Xtrinsic silicone inks; Spectromatch Ltd., Hampton, UK) were applied to replicate subtle variations in skin hue, surface texture, and pigmentation. The completed prosthesis was then tried on the patient's right hand, demonstrating good adaptation, comfort, and aesthetic harmony with the contralateral hand, as shown in Figure 15.

Final Insertion

**Figure 16:**
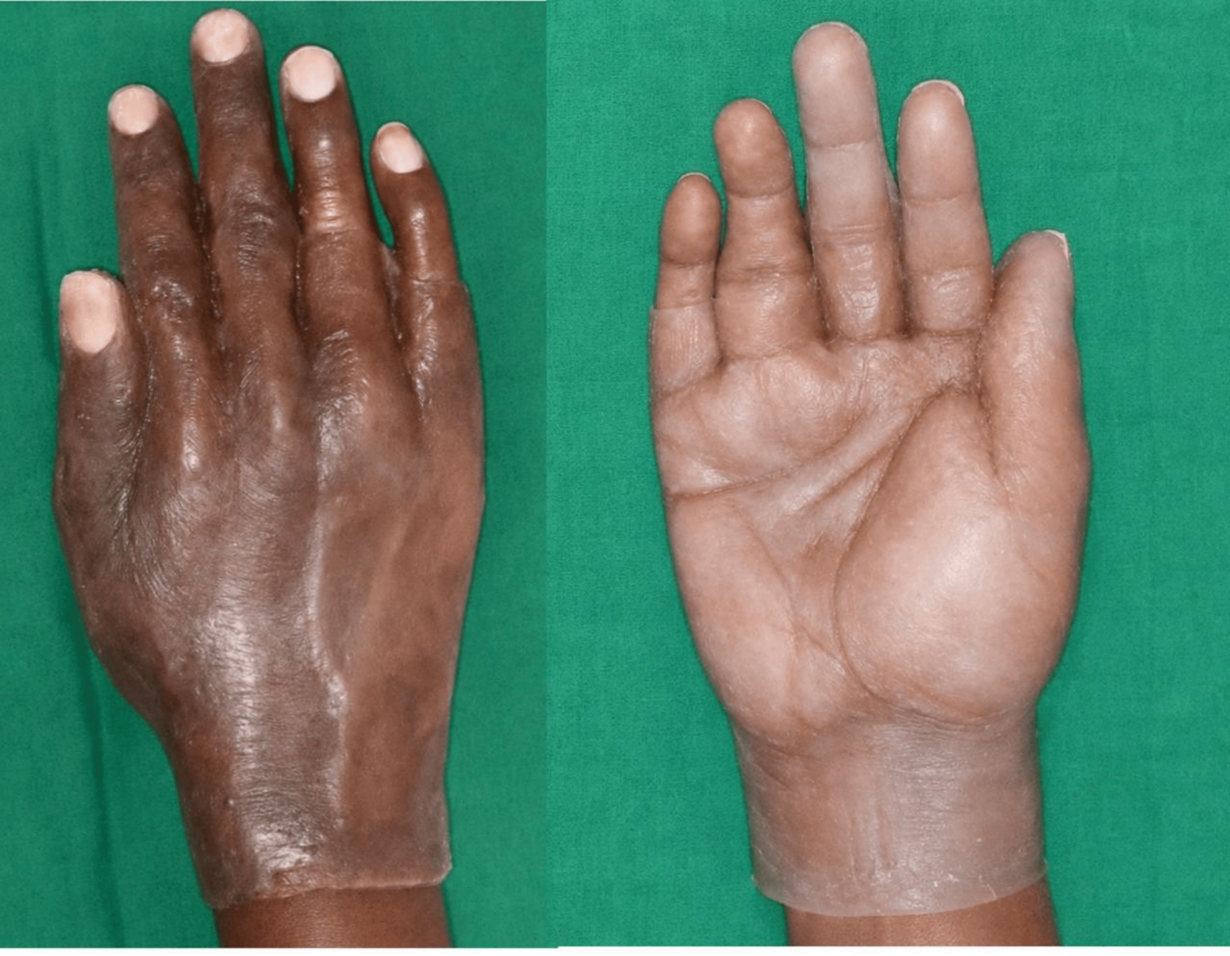
The completed silicone finger prosthesis was inserted, and the patient was instructed on its use, care, and maintenance

The final prosthesis was placed on the patient's hand. The patient was instructed about the use and maintenance of the prosthesis. The patient was advised to clean the prosthesis daily with mild soap and lukewarm water, pat it completely dry, and store it in a ventilated container away from heat and sunlight. Excessive bending, stretching, or contact with oils should be avoided. Periodic follow-up visits were recommended to evaluate the fit, color stability, and surface condition of the prosthesis and to perform any necessary adjustments. The final prosthetic outcome following insertion is shown in Figure 16.

## Discussion

Traumatic amputation of the thumb and digits results in significant functional impairment and psychological distress. Rehabilitation aims not only to restore function but also to improve aesthetics and patient confidence [4]. In both cases presented, a custom-fabricated silicone prosthesis provided a practical, non-invasive solution for rehabilitation.

In the present cases, an alternative impression technique was adopted. The patient's hand was positioned comfortably on a flat table surface, ensuring a natural, relaxed posture from the elbow down to the hand during impression making. This approach provided better positioning control, minimized distortion, and allowed a more accurate reproduction of hand contours.

Room temperature vulcanizing (RTV) silicone (Part A of Part B of the M511, Technovent, Bridgend, UK) was selected for prosthesis fabrication. This material was preferred because its Shore hardness value closely matches that of silicones used in glove and hand prostheses, offering optimal flexibility, elasticity, and skin-like texture. RTV silicone also possesses excellent tear resistance, dimensional stability, and biocompatibility, making it highly suitable for long-term extraoral use. Moreover, it allows both intrinsic and extrinsic staining, which enables lifelike color reproduction and fine-tuning of aesthetic detail.

Functional (mechanical) prostheses were not selected in these cases because the amputations were isolated and did not involve major joint loss or complex motion requirements. The primary concern was aesthetic restoration rather than movement or grip function. In such scenarios, a passive silicone prosthesis offers a more reliable, lightweight, and cost-effective solution [5]. In addition, the presence of an adequate residual stump was an important factor contributing to the retention and stability of the prosthesis. The remaining hard and soft tissue support facilitated proper adaptation of the silicone prosthesis and enhanced the overall esthetic outcome.

The custom-made RTV silicone prostheses fabricated in these cases effectively restored the natural contour of the hand, camouflaged the defect, and significantly improved the patients' confidence and social acceptance.

## Conclusions

Custom-made silicone prostheses offer an excellent aesthetic solution for patients with partial digital loss following trauma. The use of RTV silicone provided a lifelike appearance with good flexibility, comfort, and durability. This conservative, non-surgical approach effectively restored the natural hand contour and improved the patients' psychological well-being, making it a dependable option when functional prostheses or surgical reconstruction are not indicated.

## References

[1] Dillingham TR, Pezzin LE, MacKenzie EJ (2002). Limb amputation and limb deficiency: epidemiology and recent trends in the United States. South Med J.

[2] Belter JT, Segil JL, Dollar AM, Weir RF (2013). Mechanical design and performance specifications of anthropomorphic prosthetic hands: a review. J Rehabil Res Dev.

[3] Scolozzi P, Jaques B (2004). Treatment of midfacial defects using prostheses supported by ITI dental implants. Plast Reconstr Surg.

[4] Bickel KD (2007). The dorsal approach to silicone implant arthroplasty of the proximal interphalangeal joint. J Hand Surg Am.

[5] Jacob PC, Shetty KH, Garg A, Pal B (2012). Silicone finger prosthesis. A clinical report. J Prosthodont.

